# Determination of Non-Transferrin Bound Iron, Transferrin Bound Iron, Drug Bound Iron and Total Iron in Serum in a Rats after IV Administration of Sodium Ferric Gluconate Complex by Simple Ultrafiltration Inductively Coupled Plasma Mass Spectrometric Detection

**DOI:** 10.3390/nano8020101

**Published:** 2018-02-11

**Authors:** Murali K. Matta, Christopher R. Beekman, Adarsh Gandhi, Suresh Narayanasamy, Christopher D. Thomas, Adil Mohammad, Sharron Stewart, Lin Xu, Ashok Chockalingam, Katherine Shea, Vikram Patel, Rodney Rouse

**Affiliations:** 1 U.S. Food and Drug Administration, Center for Drug Evaluation and Research, Office of Translational Science, Office of Clinical Pharmacology, Division of Applied Regulatory Science, Silver Spring, MD 20993, USA; murali.matta@fda.hhs.gov (M.K.M.); christopher.beekman@fda.hhs.gov (C.R.B.); adarsh.gandhi@fda.hhs.gov (A.G.); Suresh.narayanasamy@fda.hhs.gov (S.N.); thomdc15@wfu.edu (C.D.T.); sharron.stewart@fda.hhs.gov (S.S.); lin.xu@fda.hhs.gov (L.X.); ashok.chockalingam@fda.hhs.gov (A.C.); katherine.shea@fda.hhs.gov (K.S.); vikram.patel@fda.hhs.gov (V.P.); 2 U.S. Food and Drug Administration, Center for Drug Evaluation and Research, Office of Pharmaceutical Quality, Office of Testing and Research, Division of Product Quality Research, Silver Spring, MD 20993, USA; adil.mohammad@fda.hhs.gov

**Keywords:** inductively coupled plasma-mass spectrometry, ultrafiltration, pharmacokinetics, iron, iron gluconate, nanoparticles

## Abstract

A rapid, sensitive and specific ultrafiltration inductively-coupled plasma mass spectrometry method was developed and validated for the quantification of non-transferrin bound iron (NTBI), transferrin bound iron (TBI), drug bound iron (DI) and total iron (TI) in the same rat serum sample after intravenous (IV) administration of iron gluconate nanoparticles in sucrose solution (Ferrlecit^®^). Ultrafiltration with a 30 kDa molecular cut-off filter was used for sample cleanup. Different elution solvents were used to separate each form of iron from sample serum. Isolated fractions were subjected to inductively-coupled mass spectrometric analysis after microwave digestion in 4% nitric acid. The reproducibility of the method was evaluated by precision and accuracy. The calibration curve demonstrated linearity from 5–500 ng/mL with a regression (*r*^2^) of more than 0.998. This method was effectively implemented to quantify rat pharmacokinetic study samples after intravenous administration of Ferrlecit^®^. The method was successfully applied to a pharmacokinetic (PK) study of Ferrlecit in rats. The colloidal iron followed first order kinetics with half-life of 2.2 h and reached background or pre-dose levels after 12 h post-dosing. The drug shown a clearance of 0.31 mL/min/kg and volume of distribution of 0.05 L/kg. 19.4 ± 2.4 mL/h/kg.

## 1. Introduction

Iron is an essential component of every cell in the body. Iron acts as a carrier for electrons, a catalyst for oxygenation and hydroxylation, and is necessary for cellular growth and proliferation. However, its most critical role is as a component of hemoglobin in the transport and storage of oxygen [1]. Without a sufficient supply of iron, hemoglobin cannot be synthesized and the number of erythrocytes in the blood cannot be maintained at an adequate level [2]. Iron supplements are widely administered to treat iron deficiency anemia, particularly in chronic diseases such as kidney disease [1], heart failure [3] or inflammatory bowel disease [4]. In these disease conditions, intravenous (IV) iron colloidal products are being used to treat serious iron deficiency anemias particularly, those requiring dialysis.

Iron colloidal products are iron carbohydrate complexes consisting of a mineral core, composed of polynuclear iron (III)-hydroxide surrounded by a carbohydrate ligand [5]. The main function of the ligand is to stabilize the complex and to protect it against further polynuclearization. In the first step of drug uptake, the stable iron-carbohydrate complexes are taken up by macrophages of the reticuloendothelial system (RES) [5] followed by fusion of the endosome with a lysosome releasing iron from the complex. The Fe^2+^ generated is transported by the divalent metal transporter 1 (DMT1) across the endolysosomal membrane to enter the labile iron pool within the macrophage cytoplasm. From there, it can be incorporated into ferritin and remain transiently stored within the macrophage or can be transported out of the macrophage by the transmembrane protein ferroportin (as Fe^2+^). The exported Fe^2+^ is immediately oxidized by ceruloplasmin to Fe^3+^ which is sequestered by transferrin for transport in the serum to sites of utilization, for example, in the bone marrow for hemoglobin synthesis or in the liver for storage in ferritin.

Upon IV administration of iron colloidal products, depending on the dose, there could be a saturation of the blood transporter protein, transferrin, resulting in circulating non-transferrin protein bound (Fe^3+^) (NTBI) iron in serum. This NTBI is potentially harmful due to its ability to form free radicals, thereby inducing oxidative stress and cellular toxicity [6,7]. Hence, it is very useful to monitor the real NTBI, or “free iron”, in circulating blood to evaluate the possibility of toxicity upon IV administration of iron colloidal products. Accurate measurement of NTBI is difficult in the presence of other iron sources like transferrin bound iron (TBI) and drug bound iron (DI). DI is the iron that forms the nanoparticle core encapsulated inside the carbohydrate shell. DI is stable in serum with particle uptake and iron extraction facilitated by macrophages, as described above [5,8]. While many analytical methods are available in the literature for the determination of iron [8,9,10,11,12,13,14,15,16,17,18,19,20], no suitable analytical method was available for accurately measuring all iron forms including total iron (TI), TBI, NTBI, and DI in a pharmacokinetic rat study. A few discrete methods were reported for determination of free iron and TBI. Kolb et al. [17] compared several available methods for measurement of free iron, of which five were based on detection with iron chelators and one assay measured redox-active iron using bleomycin. However, it was evident from reports in the literature that the nature and concentration of the chelating agent influenced the measurement of free iron [17]. Internal unpublished studies suggested that in the presence of DI, bleomycin incubation requirements resulted in release of iron from the drug and caused the over estimation of NTBI. 

The very small sample volumes obtained in serial sampling of rats preclude the use of other analytical methods for determination of TBI or and total iron [18,19] that either require larger sample volumes and/or do not allow concurrent quantification of NTBI and DI. To support a rat pharmacokinetic study, an analytical method capable of quantifying NTBI, TBI, DI and TI in small sample volumes was required. To date no suitable analytical method had been described in the literature to accurately measure the all these fractions in small sample volumes. Hence, the authors developed a simple and robust filtration technique capable of separating NTBI, TBI, and DI species (fractions) with a separate simple measure of TI. Iron concentration of all fractions and TI were then determined by inductively coupled plasma mass spectrometry (ICP-MS). During method development, the authors investigated the impact of a commonly employed chelator and bleomycin incubation conditions on estimate of NTBI to inform a method less biased toward NTBI over estimation. This less biased and validated method was then effectively applied for quantification of all forms of iron species in a rat pharmacokinetic study.

## 2. Materials and Methods

### 2.1. Chemicals

All the chemicals used were of analytical reagent grade. Nitrilotriacetic acid (NTA), sodium thioglycollate (TGA), BPP3-morpholinopropane-1-sulfonic acid, 3-(*N*-morpholino) propane sulfonic acid (MOPS), ascorbic acid, sodium dithionite, holo-transferrin, DNA from calf thymus, bleomycin sulfate, magnesium chloride, thiobarbituric acid, butanol, hydrochloric acid, bovine serum albumin and magnesium chloride were purchased from Sigma Aldrich (St. Louis, MI, USA). Formic acid was procured from Fluka (St. Louis, MI, USA). Amicon^®^ Ultra 0.5 mL filters (30 kDa) for protein purification and concentration were purchased from EMD Millipore (Billerica, MA, USA). Ultra-pure nitric acid was obtained from Fisher Scientific (Waltham, MA, USA). Ferrlecit^®^. Two different standard solutions of iron (Fe) (1000 ppm) were purchased from two separate sources Perkin Elmer (Shelton, CT, USA) and High Purity Standards (Charleston, SC, USA). A solution of 1000 ppm Indium (In) was purchased from Perkin Elmer (Shelton, CT, USA). Commercial drug product was obtained from a retail pharmacy. 

### 2.2. Instrumentation

A Perkin Elmer (Shelton, CT, USA) Nexlon 300D inductively coupled plasma mass spectrometry (ICP-MS) was used for analysis. A Synergy MX, BioTek, spectrophotometer (Winooski, VT, USA), was used for colorimetric experiments. A Heraeus Multifuge X1R (Thermo Scientific, Waltham, MA, USA) centrifuge was employed in the preparation of the serum ultra filtrates. 

### 2.3. Proposed Bioanalytical Method

The proposed bioanalytical method has three different steps: ultrafiltration, sample digestion of filtered samples and ICP-MS analysis.

#### 2.3.1. Ultrafiltration Method

**Figure 1:**
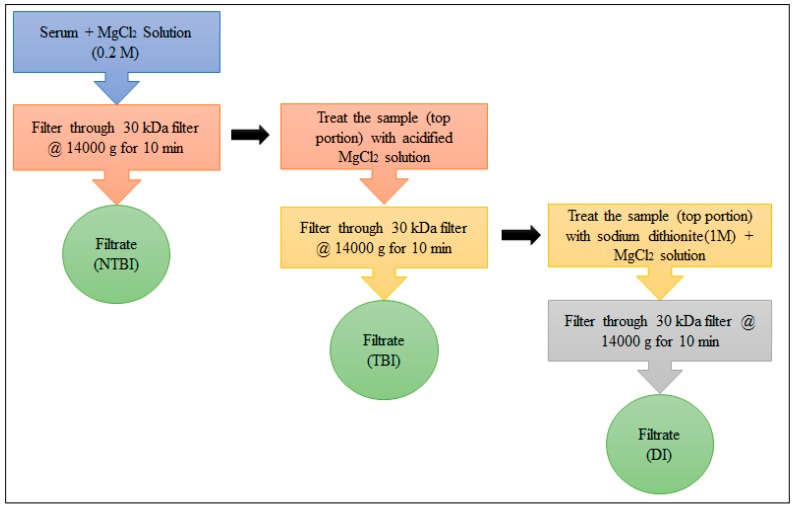
Detailed methodology of ultra-filtration procedure.

A simple ultrafiltration technique was used with a modification of the method proposed by Kolb et al. [17]. Serum samples were frozen at −20 °C until time of analysis and thawed before use. An aliquot of 25 μL of serum was mixed with 225 μL of magnesium chloride solution (0.2 M) and allowed to stand at room temperature for 20 min. The solution was then ultra-filtered using Amicon ultra filters (MW 30,000 Da cutoff), with an applied centrifugal force of 14,000 *g* for 10 min to separate the resulting free iron as ferric chloride complex from TBI and DI. As a second step, the same filter was treated with 200 µL of magnesium chloride solution (0.5% formic acid) and the filter was again centrifuged at 14,000 *g* for 10 min to separate TBI from the filter while retaining DI. As a final step, the same filter was treated with 50 µL sodium dithionite (1 M) and 150 µL of magnesium chloride solution. The solution was ultra-filtered at 14,000 *g* for 10 min to elute the DI into the filtrate. A separate sample was prepared for the estimation of total iron by microwave digestion. The detailed flow chart of sample preparation is represented in Figure 1.

#### 2.3.2. Sample Digestion

Each fraction of filtrate was pre-digested in 4% nitric acid in microwave before ICP-MS analysis. The parameters of the microwaving cycle were as follows: power 1200 W; temperature, 200 °C; pressure, 200 psi; control style, ramp to temperature; hold time, 20 min; and stirring, off. A set of quality control (QC) samples were prepared to evaluate the efficiency, accuracy and reproducibility of the proposed ultrafiltration steps. Iron is an endogenous compound observed even in blank serum samples. Hence, 5% bovine serum albumin (BSA) solution in water was used as a surrogate matrix for the preparation of quality control (QC) samples. The QC samples at four levels were prepared by cassette spiking of elemental iron, holo-transferrin and drug formulation reflecting the anticipated pharmacokinetic samples after IV administration of iron colloidal formulation. These QC samples were subjected to filtration as mentioned earlier and analysed by the ICP-MS method after microwave digestion of each sample.

#### 2.3.3. ICP-MS Method

The optimized operating conditions used for ICP-MS are summarized in Table 1. A peristaltic pump was used to deliver the samples (0.1 mL/min) to the nebulizer, which in turn converted the sample into a spray mist using argon (Ar) gas. Instrumental settings were optimized daily prior to analysis using a tuning solution consisting of Boron (B), Barium (Be), Iron (Fe), Indium (In), Lithium (Li), Magnesium (Mg), Lead (Pb) and Uranium (U) to establish system suitability for daily operation. Automated adjustments were made for torch alignment, detector voltage and ion lens voltages for optimized resolution, sensitivity, and stability across a broad range of atomic masses. The doubly charged ion/charge ratio (Ce^2+^ 69.95 to Ce 139.90) and oxide ratio (CeO^+^ 155.90 to Ce 139.90) were also monitored and were maintained below 3% and 2.5% (intensity) respectively. The PrepFast system was interfaced to the ICP-MS and used to auto-dilute the stock solutions to generate the calibration curves and QCs samples. The 30 ppb indium (In) solution was also mixed in-line by the Prep-Fast during the analysis as internal standard to all the analyzed samples and standards. The method was evaluated for its selectivity, sensitivity, linearity, precision and accuracy. 

### 2.4. In Vitro Incuation Studies to Evaluate Advantages of Proposed Method

In the literature, two different methods were described for the measurement of NTBI in serum samples, one method involves the use of chelating agent (NTA) in the ultrafiltration step [17] and the other one is incubating with non-chelating agent (bleomycin) [20] followed by colorimetric detection. However, these methods were not thoroughly evaluated for accuracy in presence of DI, hence a set of incubation studies were designed to evaluate the effect of assay conditions/reagents on the artifacts of the assay measurements and the efficiency of the proposed ultrafiltration method using MgCl_2_ solution over the reported methods for NTBI measurements. 

Incubation studies with chelating agent (NTA): Studies were conducted to assess the impact of incubation with chelating agent (NTA) and non-chelating agent like bleomycin on measured free iron concentrations. The potential iron leaching effect of NTA on iron colloidal formulation was evaluated by incubating the iron colloidal drug formulation (100 µg/mL in 5% BSA) with 0.8 M of NTA and collecting samples after 15, 30, 60 and 120 min. These results were then compared to aliquots of the same samples used with magnesium chloride solution as per the proposed ultrafiltration method. The concentration of all these samples were measured by the reported spectrophotometric method [17] and validation results were presented in Table S1. 

Incubation studies with nonchelating agent (Bleomycin): The bleomycin assay requires incubation of the samples with bleomycin at 37 °C for two hours. Experiments were designed to evaluate the effect of high temperature and bleomycin on measured free iron concentrations. To evaluate the impact of bleomycin incubation on measured free iron concentrations in the bleomycin assay, serum samples containing all iron species (harvested 15 min after IV drug bolus) were obtained and incubated at 37 °C for two hours with and without bleomycin. To assess the impact of temperature aliquots of the same samples were kept at room temperature with and without bleomycin. All samples were passed through the 30 kDa ultra filtration step using MgCl_2_ as described in proposed method. These ultra filtrates were used for the bleomycin assay [20], which was validated at in-house and results were reported in Table S2. The effects of bleomycin incubation on free iron measures in the presence of holo-transferrin iron and drug iron were investigated in vitro.

### 2.5. Pharmacokinetic Study in Sprague Drawley Rats

Animals: All animal experiments were conducted under an approved protocol and oversight of the Food and Drug Administration’s White Oak Federal Research Center Institutional Animal Care and Use Committee. Animal research was conducted in accordance with the Guide for the Care and Use of Laboratory Animals, 8th Edition (NRC 2011). Dual cannulated male Sprague Dawley rats approximately 300–350 g were purchased from Taconic Farms (Derwood, MD, USA). Each rat had a cannula placed in the right jugular vein and in the left femoral vein. Animals were kept on a 12-h light cycle and received food and water ad libitum. All rats were acclimated for a minimum of 7 days prior to initiation of experiments. 

Pharmacokinetic study design: The developed method was used to analyze serum samples from a rat pharmacokinetic study. Pharmacokinetic studies were conducted in 16 male Sprague Drawly rats dosed at 40 mg/Kg to assure free iron measurement at transferrin saturation. Measuring of transferrin saturated levels of free iron would assure sufficient free iron concentrations for measurement and in the future, would allow comparison of free iron levels derived from different formulations or products. Blood samples (0.20 mL) were withdrawn at 0, 0.08, 0.25, 0.5, 1, 2, 3, 4, 8 and 24 h post-IV administration. Sixteen male rats with femoral and jugular vein were obtained from (Taconic Biosciences, New York, NY, USA). Each rat was dosed through the femoral vein and sampled through the jugular cannula. After dosing, two serial samples were taken from groups of four rats so that one group of four were sampled at 5 and 15 min, the next group at 30 and 60 min, and so on until the 4 groups of 4 rats had provided samples at 8 time points. Terminal samples were acquired from all 16 rats at 24-h post-treatment. Blood was collected into 1 mL syringes, transferred to 1.5 mL plastic tubes and centrifuged for 10 min at 3000 rpm after standing at room temperature for 60 min; serum was decanted and stored at −80 °C. These samples were subjected to ICP-MS analysis after ultrafiltration and microwave digestion. The pharmacokinetic parameters were calculated by using Phoenix^®^ (version 7.0.0, Princeton, NJ, USA) software using the non-compartmental method. Concentrations below the limit of quantitation were treated as zero pharmacokinetic calculations. The mean concentration values from all sixteen animals of pre-dose were subtracted from individual values of each group to eliminate the base level (endogenous) concentrations. After subtracting, any negative values at 24 h were considered as zero for PK calculations. The area under the plasma concentration versus time curve (AUC) was calculated using the linear trapezoidal method. When appropriate, the terminal elimination phase of the pharmacokinetic profile was estimated for the drug concentrations based on the best fit using at least the last three observed concentration.

## 3. Results

### 3.1. Proposed Bioanalytical Method

The results of precision and accuracy of the method are described in the following sections.

#### 3.1.1. Ultrafiltration Method and Sample Digestion

The precision accuracy data of QC samples reflect the reproducibility of the ultrafiltration and digestion steps. The QC samples were ±20% of the true values with % relative standard deviation (RSD) of less than 15%. The detailed accuracy and precision values were represented in Table 2.

#### 3.1.2. ICP-MS Method

The accuracy and precision values of ICP-MS detection were measured on three different days and the results were ±20% of the true values with % RSD of less than 15%.

(i) Selectivity

A fundamental challenge in the development and optimization of any analytical method is the matrix interference. This is especially important when matrices have endogenous levels of the analyte of interest. ICP-MS reduces many of the traditional matrix issues encountered by atomic spectroscopy methods. During method development, selectivity was established for both neat and biological matrices. The method was selective and didn’t give any interference in blank samples.

(ii) Sensitivity

Samples containing elemental iron at concentrations of 0, 5, 10, 50, 100, 200, and 500 ng/mL were prepared in 2% nitric acid containing magnesium chloride solution (0.2 M). The instrument responses (counts) of iron-containing samples were compared to the blank. The lowest iron concentration of 5 ng/mL, was selected as reportable lower limit of quantitation (LLOQ) as signal to noise ratio was more than fivefold. 

(iii) Linearity

An iron calibration curve was generated using one stock solution which was auto diluted using PrepFast to generate six calibration standards in 2% nitric acid containing magnesium chloride, representing the biological samples. The concentrations of the iron calibration standards include 5, 10, 50, 100, 200, and 500 ng/mL. Each sample was analyzed three times, and the average of the three replicates was calculated (detailed data not shown). The percent RSD values are within the acceptable range of ±15% at each tested concentration. In addition, regression (*r*^2^) values were more than 0.995 for all tested curves. The linearity results were presented in Table S3.

(iv) Precision and Accuracy

The precision and accuracy of the method was evaluated over the range of the calibration curve in quality control samples. The precision and accuracy of a low, medium, and high iron concentration was determined using three measurements per concentration. The precision and accuracy values were listed in Table 3.

### 3.2. In Vitro Incubation Studies

**Figure 2:**
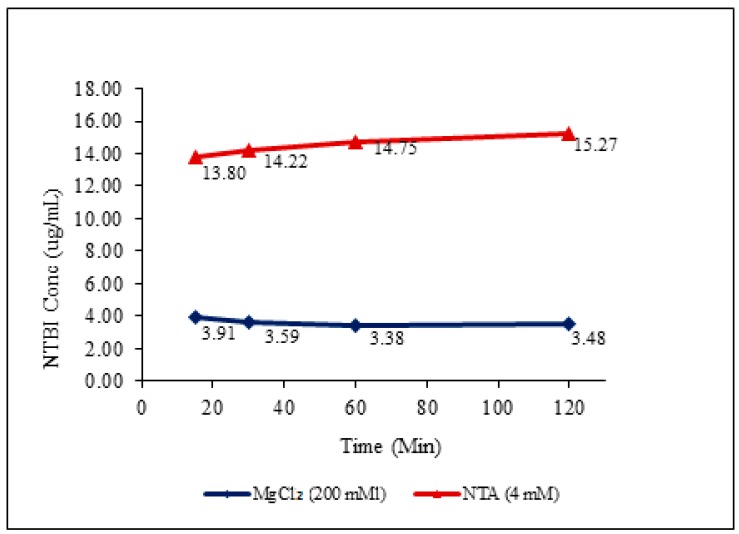
Chelation effects of nitrilotriacetic acid (NTA) and MgCl_2_ on Iron colloidal drug with time.

The effect of NTA on drug formulation was evaluated over 120 min including 15, 30, 60 and 120 min sampling (Figure 2). Samples were subjected to ultrafiltration sample cleanup before spectrophotometric measurement. Those samples incubated with NTA showed significantly higher free iron concentration (3 to 4-fold) compared to MgCl_2_ samples tested from all time points and it was also noted that concentrations were increasing with time of incubation (Figure 2). 

**Figure 3:**
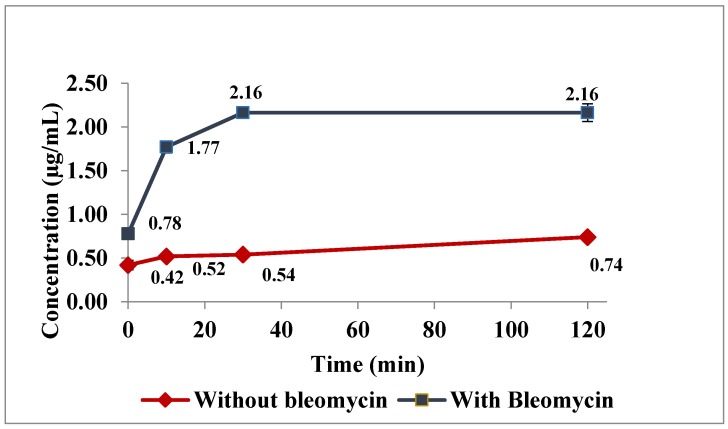
Chelation effect of Bleomycin in in-vivo samples.

Similar observations were noted in bleomycin incubation studies using serum of recently treated animals as well as in vitro studies. Figure 3 shows the difference in recorded free iron concentration caused by incubation at 37 °C with and without bleomycin. Bleomycin exposure resulted in immediately higher (≈80% higher) free iron measurements compared to incubation at the same temperature without bleomycin. Over a two-hour incubation, this difference escalated to 300–400 percent higher with bleomycin. It should be noted that even without bleomycin free iron measurement increased approximately 80 percent over the two hours. 

**Figure 4:**
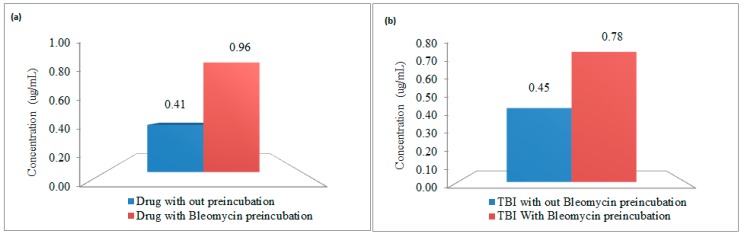
Chelation effect of bleomycin on (**a**) Iron colloidal drug; (**b**) Transferrin bound iron.

The bleomycin incubation results in elevated free iron measures in the presence of drug and transferrin-bound iron (Figure 4). 

### 3.3. Pharmacokinetic Study in Sprague Drawley Rats

**Figure 5:**
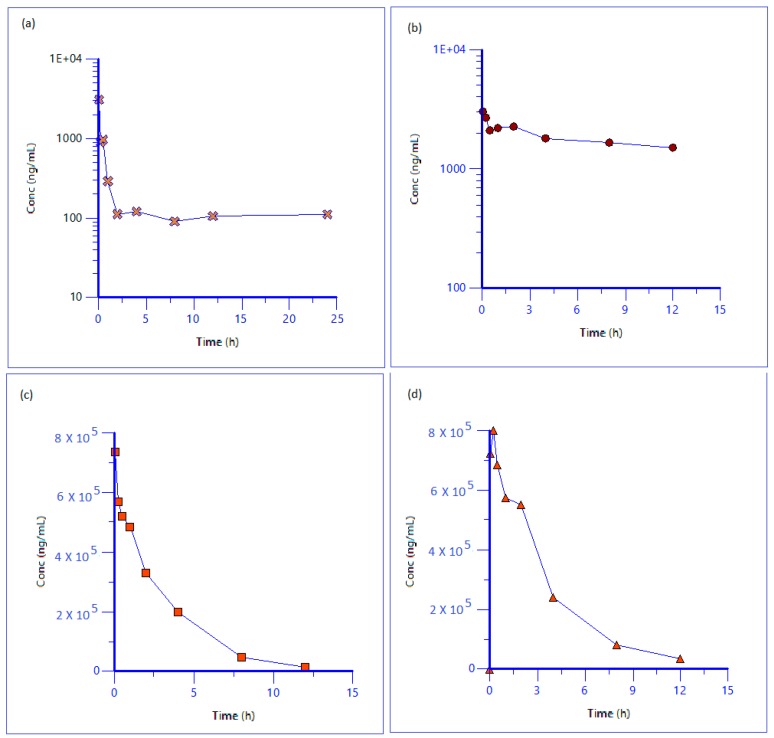
Concentration–time profile of different species of iron (**a**) Free iron (NTBI); (**b**) Transferrin bound iron (TBI); (**c**) Drug bound Iron (DI); (**d**) Total Iron (TI) upon iv bolus (40 mg/kg) administration of colloidal iron formulation in male Sprague Dawley Rats.

Intravenous administration of iron colloidal formulation as a single bolus dose to male Sprague-Dawley rats at 40 mg/kg was well tolerated and all rats remained alert, active and responsive throughout the study. No clinical or anatomical pathology parameters were evaluated in this study. Blood samples were obtained prior to dose and 0.08, 0.25, 0.5, 1, 2, 3, 4, 8, 12, 24 h post-IV administration. Approximately, 600 ng/mL of FI (which includes elemental iron and iron bound to smaller peptides/proteins/other molecules/salts having molecular weight less than 30 kDa) was measured in pre-treatment serum samples with increased concentrations upon post-treatment with maximum serum concentration (*C*_max_) of 3500 ng/mL at time 5 min of post dose. Mean serum concentration–time profiles of TI and DI decreased in a first order manner following a single IV dose of 40 mg/kg. Drug iron reached background or pre-dose levels 12 h after dosing. The detailed pharmacokinetic parameters of drug iron are listed in Table 4. The concentration–time profiles of all forms of iron are graphically represented in Figure 5.

## 4. Discussion

The reproducibility of ICP-MS detection was assessed by running a complete validation. The reproducibility and accuracy of the ultrafiltration step was thoroughly evaluated on three different days as a part of the validation. The method measured all iron species (TI, DI, TBI, NTBI) in 25 µL of serum with a sensitivity of 5 ng/mL. Although NTBI is a small fraction of the serum iron, its measurement is considered essential since it represents iron that is most capable of participating in reduction/oxidation (redox) reactions to yield reactive oxygen species and potentially cause tissue damage. Most of the available methods for the estimation of NTBI use varying concentrations of chelating agents like oxalate, EDTA, and NTA. Literature demonstrates that the estimation of NTBI in the same sample varies on the type and concentration of agent used [18]. Upon NTA addition, there was a bleeding of iron from the drug formulation that was confirmed by comparing the free concentrations obtained from NTA elution against MgCl_2_ elution samples. The results clearly reflect the bleeding effect of NTA followed by slow leaching. A similar experiment was performed with holo-transferrin, where the bleeding effect was not significant (data not shown), indicating that iron was not being leached from TBI. These findings confirm that chelating agents can lead to overestimation of free iron.

Hence, a method was developed devoid of any chelating agent to minimize these artifacts. The initial MgCl_2_ filtration step yields a filtrate that most accurately reports immediately available charged iron. The mechanism of action of MgCl_2_ is unclear, however the hypothesis is the MgCl_2_ reduces the ionized state of iron (Fe^3+^) to unionized state of iron (Fe^2+^), which can pass through the ultrafilter membrane [21]. Moreover, the efficiency of this solution was thoroughly evaluated during initial method development over other solvents like saline, water, and so on. The subsequent denaturing of the protein captured on the filter and elution of iron held in that protein includes TBI and potentially iron that was more loosely bound to other proteins. The last step of ultrafiltration involves, treating the sample with sodium dithionate which ruptures the nanoparticles and releases the iron encapsulated in carbohydrate coating [18]. Importantly, the developed method is capable of estimating DI, a critical component of serum iron for hours immediately following administration of colloidal iron products. Direct NTBI measurement by bleomycin assay produced significantly higher NTBI concentrations in rat pharmacokinetic study samples. With the bleomycin assay, NTBI values were consistently much higher at all time points and especially the critical early time points. These results indicate that bleomycin pulls iron either from transferrin bound or drug bound iron or both. This leaching process increased with time at 37 °C up to 30 min and was then level up to 2 h. Notably, temperature alone also increased NTBI, as observed in samples not incubated with bleomycin that had increased NTBI with time. To characterize the leaching effect, incubation experiments were performed with iron colloidal drug and holo-transferrin. Results showed that the bleomycin had the ability to bleed iron from colloidal iron drug and to a lesser degree from transferrin dependent upon concentration of TBI. Collectively, these data suggest that existing methods may over estimate NTBI at early time points by pulling iron from drug and at later time points by acquiring iron from the increased pool of highly saturated TBI and demonstrate the need for this novel method. 

After completing the preliminary evaluation of the reproducibility of the proposed ultrafiltration method, the method was validated for its accuracy and reproducibility by three precision and accuracy batches and then successfully applied in a rat pharmacokinetic study. The colloidal iron followed first order kinetics with half-life of 2.2 h and reached background or pre-dose levels after 12 h of post-dosing. The drug had clearance value of 0.31 mL/min/kg and volume of distribution of 0.05 L/kg. After dosing, the NTBI and TBI levels rose rapidly and dropped to background or pre-dose levels within 4 h.

## 5. Conclusions

For the first time, a simple and reproducible ultra-filtration technique with sensitive detection was developed for the simultaneous quantification of NTBI, TBI, DI and TI within a very small sample volume. The efficiency of the method was evaluated against reported methods, especially for NTBI, demonstrating that the proposed method had significant advantages by avoiding over estimation of free iron concentrations. This method can be adopted for clinical study sample analysis and may serve to evaluate true NTBI concentration with better sensitivity and minimal bias particularly in the presence of iron colloid drug products. The method was successfully applied to determine the pharmacokinetic parameters of all different iron fractions from very low sample volumes in a study of Ferrlecit in rats. The colloidal iron followed first order kinetics with half-life of 2.2 h and reached background or pre-dose levels 12 h post-dosing. The drug had clearance of 0.31 mL/min/kg and volume of distribution of 0.05 L/kg. The drug also demonstrated transient elevations is NTBI and was used for determination of intravenous sodium ferric gluconate complex in a biodistribution study [22].

## Figures and Tables

**Table 1:** Inductively coupled plasma mass spectrometry (ICP-MS) operating conditions and acquisition parameters.

**ICP Parameters**	
RF Power	1600 W
Plasma gas flow rate	18.0 L/min
Auxiliary gas flow rate	1.2 L/min
Nebulizer gas flow rate	1.0 L/min
Spray chamber	Peltier-cooled (2 °C) baffled quartz cyclonic spray chamber
Torch	Quartz
Sampler and skimmer cones	Platinum
Hyper skimmer cones	Nickel
**Mass Spectrometer Parameters**	
Resolution	0.7 amu at 10% peak maximum
Dwell time	50 ms
Sweeps	20
Readings	1
Replicates	3
Autolens	ON
Internal Standard	In (115 amu)
Mode of detection	Kinetic Energy Differentiation (KED)

**Table 2:** Accuracy and precision data of Ultrafiltration method.

QC	Nominal Concentration (ug/mL)	Calculated Concentration Mean ± SD (ug/mL)	Accuracy (%)	% RSD
NTBI				
Low QC	2.2	1.8 ± 0.1	85	8.0
Mid QC	3.3	3.6 ± 0.3	109	9.3
High QC	3.3	7.3 ± 0.2	106	2.6
TBI				
Low QC	6.0	7.0 ± 0.1	117	1.4
Mid QC	10.0	10.4 ± 0.1	104	1.1
High QC	18.0	18.3 ± 0.7	101	3.7
DI				
Low QC	50.0	56.3 ± 2.3	113	4.0
Mid QC	100.0	107.3 ± 1.8	107	1.7
High QC	250.0	249.8 ± 5.3	100	2.1

**Table 3:** Accuracy and precision Data of ICP-MS analytical method.

QC	Nominal Concentration (ng/mL)	Calculated Concentration (ng/mL)	Calculated Concentration Mean ± SD (ng/mL)	Accuracy	% CV
QC-1	5	4.6	4.8 ± 0.4	96	9.1
		4.5			
		5.3			
QC-2	50	48.2	48.6 ± 0.6	97	1.3
		48.3			
		49.3			
QC-3	200	196.2	196.7 ± 0.4	98	0.2
		197.0			
		196.8			
QC-4	500	588.7	489.5 ± 4.7	98	1.0
		494.6			
		485.3			

**Table 4:** Pharmacokinetic parameters.

Type of Iron	Half Life (h)	V_ss_ (L/kg)	MRT (h)	AUC_last_ (h·ng/mL)	Cl (mL/min/kg)
Free Iron (NTBI)	-	-	-	3552.2	-
Transferrin Bound Iron	-	-	-	21,880.3	-
Drug Iron	2.2	0.05	3.0	2,120,035.7	0.3
Total Iron	-	-	-	2,886,456.9	-

## Supplementary Materials

The following are available online at http://www.mdpi.com/2079-4991/8/2/101/s1, Table S1: Precision and accuracy data of spectrophotometric method; Table S2: Precision and accuracy data of Bleomycin assay; Table S3: Linearity data of ICP-MSICP-MS method.
