# Supplementary material for: Determination of Non-Transferrin Bound Iron, Transferrin Bound Iron, Drug Bound Iron and Total Iron in Serum in a Rats after IV Administration of Sodium Ferric Gluconate Complex by Simple Ultrafiltration Inductively Coupled Plasma Mass Spectrometric Detection
Source: Nanomaterials (Basel). 2018 Feb 11;8(2):101. doi: 10.3390/nano8020101 (PMC5853732; doi:10.3390/nano8020101)
Supplement: Supplementary file 1 [file nanomaterials-08-00101-s001.docx]

**Table S1:** Precision and accuracy data of spectrophotometric method,

| Quality control | Run | Concentration found  Mean±SD (µg/mL) | Precision (%) | Accuracy  (%) |
| --- | --- | --- | --- | --- |
| **Intra–day variations (*n*=3 at each concentration)** | | | | |
| LLOQ | | 2.25 ± 0.03 | 1.47 | 112.3 |
| LQC | | 5.07 ± 0.04 | 0.74 | 92.3 |
| MQC1 | | 12.35 ± 0.19 | 1.57 | 102.9 |
| MQC2 | | 22.83 ± 0.34 | 1.48 | 95.1 |
| HQC | | 39.57 ± 0.34 | 0.87 | 98.9 |
| **Inter–day variations (*n*=6 at each concentration)** | | | | |
| LLOQ | | 2.34 ± 0.11 | 4.67 | 117.0 |
| LQC | | 5.11 ± 0.06 | 1.13 | 92.9 |
| MQC1 | | 12.43 ± 0.19 | 1.49 | 103.6 |
| MQC2 | | 22.92 ± 0.40 | 1.75 | 95.5 |
| HQC | | 40.25 ± 0.87 | 2.16 | 100.6 |
| Nominal concentrations of LLOQ, LQC, MQC1, MQC2 and HQC are 2.00, 5.50, 12.00, 24.00 and 40.00 µg/mL, respectively | | | | |

**Table S2:** Precision and accuracy data of Bleomycin assay

| **Added conc. (µg/mL)** | **Recovered conc. (µg/mL)** | **% Nominal** |
| --- | --- | --- |
| 0.1 | 0.09 | 89.59 |
| 0.5 | 0.492 | 98.35 |
| 1.0 | 1.007 | 100.74 |

**Table S3a**– **Method validation (linearity) parameters for the ICP-MS analytical method**

| **Day** | **Range (ppb)** | **Slope** | **Intercept** | **R^2^** |
| --- | --- | --- | --- | --- |
| Day 1 | 5-500 | 0.0142 | -0.0273 | 0.9978 |
| Day 2 | 5-500 | 0.0128 | -0.0301 | 0.997 |
| Day 3 | 5-500 | 0.0138 | -0.0303 | 0.9969 |

**Table S3b– Precision and accuracy data for the ICP-MS analytical method**

| **Nominal Conc. (ppb)** | **Intra-day (n=5)** | | | **Inter-day (n=3)** | | |
| --- | --- | --- | --- | --- | --- | --- |
|  | **Mean Conc. (ppb)** | **Accuracy (%)** | **Precision (RSD %)** | **Mean Conc. (ppb)** | **Accuracy (%)** | **Precision (RSD %)** |
| 5.00 | 5.51 | 110.21 | 1.74 | 5.83 | 116.60 | 4.75 |
| 10.00 | 10.27 | 102.73 | 1.94 | 10.44 | 104.43 | 2.55 |
| 100.00 | 95.43 | 95.43 | 0.93 | 97.08 | 97.08 | 3.33 |
| 500.00 | 528.82 | 105.76 | 0.70 | 554.16 | 110.83 | 4.20 |
